# Extended Spherical Diffusion Theory: Electrochemiluminescence Imaging Analysis of Diffusive Molecules from Spherical Biosamples

**DOI:** 10.1021/acs.analchem.4c03167

**Published:** 2024-11-19

**Authors:** Kosuke Ino, Miyu Mashiko, Yusuke Kanno, Yeyi Tang, Shuzo Masui, Takasi Nisisako, Kaoru Hiramoto, Hiroya Abe, Hitoshi Shiku

**Affiliations:** † Graduate School of Engineering, Tohoku University, 6-6-11-604 Aramaki-aza Aoba, Aoba-ku, Sendai 980-8579, Japan; ‡ Institute of Integrated Research, Institute of Science Tokyo, Yokohama 226-8503, Japan; § Department of Mechanical Engineering, School of Engineering, Institute of Science Tokyo, Tokyo 152-8550, Japan; ∥ Department of Precision Engineering, The University of Tokyo, Hongo 7-3-1, Bunkyo-ku, Tokyo 113-8656, Japan; ⊥ Frontier Research Institute for Interdisciplinary Sciences, Tohoku University, Aramaki-aza Aoba 6-3, Aoba-ku, Sendai 980-8578, Japan; # Graduate School of Environmental Studies, Tohoku University, 6-6-11-604 Aramaki-aza Aoba, Aoba-ku, Sendai 980-8579, Japan

## Abstract

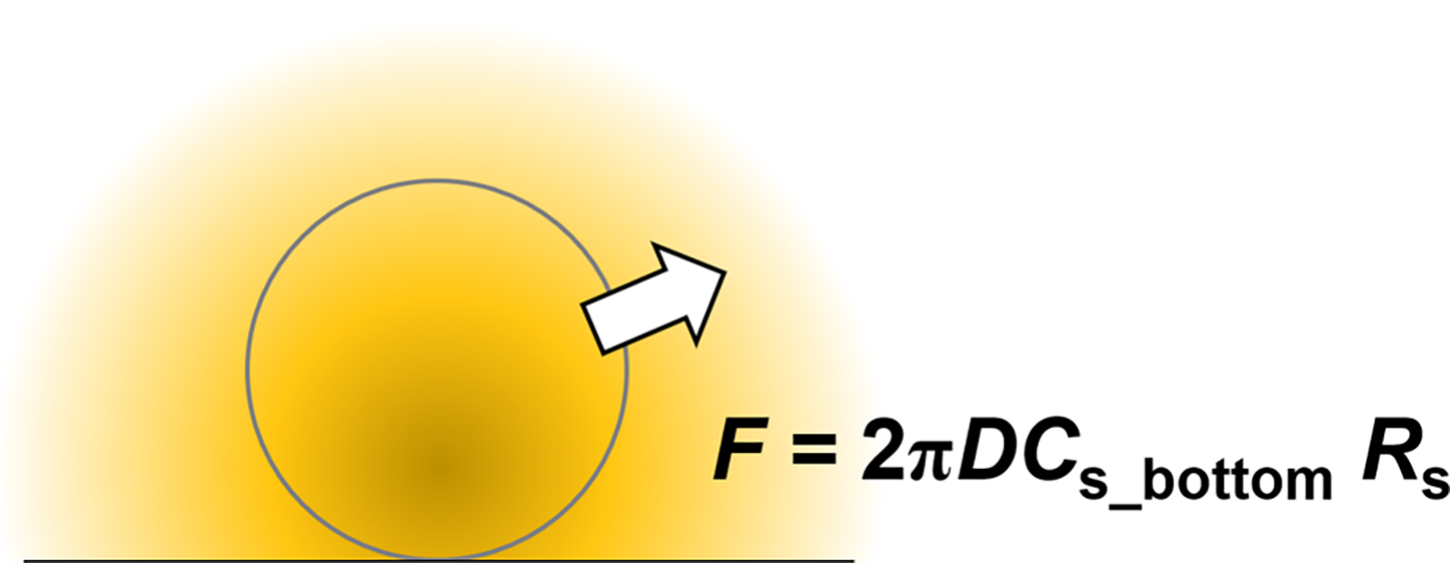

Spherical biosamples such as immunobeads, cells, and cell aggregates have been widely used in bioapplications. The bioactivity of individual spherical biosamples in highly sensitive assays and individual analyses must be evaluated in a high-throughput manner. Electrochemiluminescence (ECL) imaging was recently proposed for the high-throughput analysis of diffusive molecules from spherical biosamples. ECL imaging involves the placing of spherical biosamples on a flat electrode filled with a solution. The biosamples produce (or consume) biological/chemical molecules such as H_2_O_2_ and O_2_, which diffuse to form a concentration gradient at the electrode. The ECL signals from the molecules are then measured to obtain the concentration profile, which allows the flux to be estimated, from which their bioactivities can be successfully calculated. However, no studies on theoretical approaches for spherical biosamples on flat surfaces have been conducted using ECL imaging. Therefore, this paper presents a novel spherical diffusion theory for spherical biosamples on a flat surface, which is based on the common spherical diffusion theory and was designated as the extended spherical diffusion theory. First, the concepts behind this theory are discussed. The theory is then validated by comparison with a simulated analysis. The resulting equation successfully expresses the concentration profile for the entire area. The glucose oxidase activity in the hydrogel beads is subsequently visualized using ECL imaging, and the enzymatic product flux is calculated using the proof-of-concept theory. Finally, a time-dependent simulation is conducted to fill the gap between the theoretical and experimental data. This paper presents novel guidelines for this analysis.

## Introduction

Spherical biosamples have been used in several bioapplications; for example, microbeads are widely used for immunoassays.^1^ These microbeads are generally fabricated from polymers, metals, carbon, and hydrogels, and their surfaces are modified with capture elements such as antibodies, aptamers, and oligonucleotides. Fluorescence and electrochemical tags on individual beads are analyzed in bead-based digital immunoassays, resulting in highly sensitive assays.^2−6^ In addition to these spherical materials, cells and spherical cell aggregates, such as spheroids^7^ and organoids,^8^ are used in bioapplications such as regenerative medicine and in vitro drug assays. The cell activities of individual samples in these applications must be evaluated because of their heterogeneity.^9,10^ The cellular respiratory activity (oxygen consumption) has previously been characterized by monitoring the diffusion layer of dissolved oxygen near cells.^11−13^ Endogenous enzymes are also measured to evaluate the cellular differentiation of stem cells by monitoring the diffusion layer of enzymatic products.^14^ Thus, the analysis of individual spherical samples is an important issue for highly sensitive assays and the analysis of heterogeneous samples.

Electrochemical approaches have been proposed to analyze diffusive molecules in individual spherical biosamples. For example, scanning electrochemical microscopy (SECM) is widely used for these analyses.^15^ In SECM, a probe electrode is scanned to measure the concentration profile near the sample. Although SECM is an excellent tool, scanning samples is time-consuming and requires professional skills. Electrode array devices have been proposed to address this issue. Although these devices are useful for high-throughput analyses because of no need of scanning, their fabrication cost is high. Furthermore, the spatial resolution of the resulting electrochemical images is too low to obtain diffusive molecule concentration profiles from small biosamples. Therefore, these devices are not suitable for comprehensive high-throughput analyses.

Electrochemiluminescence (ECL) imaging has recently garnered considerable attention for use in high-throughput bioassays,^6,16−18^ including bead-based immunoassays.^6,19^ ECL is chemiluminescence induced by electrochemical reactions. The background ECL signals, as compared with those of fluorescence assays, are low because ECL does not require an external light source. ECL imaging was recently used to evaluate respiration activity,^20,21^ cellular adhesion,^22−24^ and cellular secretion.^25^ The ECL imaging of cellular spheroids involves placing the spheroids on an electrode filled with an ECL solution. Because the spheroids consume dissolved oxygen, a dissolved oxygen diffusion layer forms around them. ECL imaging provides the oxygen concentration profile for a flat electrode. The flux per spheroid (mol/s) can then be calculated by analyzing the profile on a flat plane. Thus, ECL imaging is suitable for analyzing the concentration profile of flat surfaces. Previous methods required the performance of simulation analyses. However, no theoretical approach has been used to analyze ECL imaging. A novel approach based on the spherical diffusion theory, which we designated as the extended spherical diffusion theory for a spherical biosample on a flat surface, is proposed in this study. The results obtained using this theory were compared with the simulation results to validate the theory. The glucose oxidase (GOx) activity of hydrogel beads was then measured using ECL imaging. The enzymatic product (H_2_O_2_) was visualized to obtain the concentration profile of the enzymatic products on the electrode. The profile was analyzed using the extended spherical diffusion theory to determine the flux of the enzymatic products. Finally, a time-dependent simulation was performed to fill the gap between the theoretical and experimental data. Thus, this study presents novel guidelines for the evaluation of spherical biosamples on flat surfaces.

## Materials and Methods

### Theoretical Analysis

The theory is discussed in the Results and Discussion section.

### Simulation Analysis

Three-dimensional models simulating the concentration profiles of the molecules were constructed using the COMSOL Multiphysics software (ver. 5.4; COMSOL, Inc., USA). Briefly, a spherical sample constantly produces molecules at the bottom of the model. Detailed information regarding the boundary conditions, simulation parameters, and model conditions is provided in Figure S1.

### Fabrication of Spherical Hydrogel Beads Containing GOx

Hydrogel beads containing GOx were fabricated using a microfluidic device, and the beads were used as models for spherical biosamples. The microfluidic device was fabricated by casting polydimethylsiloxane (PDMS) (SILPOT W/C, DuPont Toray Specialty Materials K.K., Japan) into a device mold prepared using a 3D printer (Anycubic Photon Mono 4 K, Shenzhen Anycubic Technology Co., Ltd., China) and an acrylonitrile-butadiene-styrene (ABS)-like resin (Anycubic ABS-Like Resin Pro 2; Shenzhen Anycubic Technology), peeling the PDMS, and bonding it onto a PDMS sheet using an atmospheric plasma treatment (PIB-20, Vacuum Device, Japan). In the microfluidic device, two #1 channels (345 μm width; 165 μm height) symmetrically branched from the one inlet were placed crosswise on the sides of a #2 channel (210 μm width; 165 μm height) to form a droplet generator. A 20 mL plastic syringe (Terumo Corporation, Japan) was filled with liquid paraffin (0.825–0.850 g/mL, FUJIFILM Wako Pure Chemical Corporation, Japan) containing 2 wt % Span 80 (Kanto Chemical Co., Ltd., Japan). The content of the syringe was then flowed into the two #1 channels at a total flow rate of 8.0 mL/h using a syringe pump (Legato 200, KD Scientific, USA). An aqueous solution mixed with 1.7 wt % GOx (170 units/mg, FUJIFILM Wako Pure Chemical), 6.8 wt % bovine serum albumin (BSA; FUJIFILM Wako Pure Chemical), and 0.9 wt % diluted glutaraldehyde (GA; FUJIFILM Wako Pure Chemical) solution was filled in a 1 mL plastic syringe (Terumo) and flowed into the #2 channel at 0.6 mL/h using a syringe pump (Legato 180, KD Scientific). Water-in-oil droplets containing GOx were generated from the flowing oil and aqueous phases. The generated droplets were pumped into a polyethylene tube (0.5 mm i.d., 1.0 mm o.d.; Hibiki Fr No. 3, Kunii Co., Ltd., Japan) and kept static for 1.5 h. This allowed the GOx and BSA to cross-link in the droplets via the GA and the gelation of the droplets. Finally, the hydrogel beads were collected and alternately washed on a nylon mesh (108 μm opening; N–No.150T, NBC Meshtec, Inc., Japan), using hexane and ultrapure water.

### ECL Imaging

8-Amino-5-chloro-7-phenylpyrido[3,4-*d*] pyridazine-1,4(2H,3H)-dione (L-012, luminol analogue) was used for ECL imaging of H_2_O_2_.^26−31^ An indium tin oxide electrode (Hiraoka Special Glass Mfg. Co., Ltd., Japan) was used as working electrode. The electrode was covered with a chamber (diameter (φ) = 4 mm, height = 1.5 mm) of PDMS (SILPOT W/C; DuPont Toray Specialty Materials K.K.) (Figure S2). The chamber was filled with an ECL solution containing 400 μM L-012 (FUJIFILM Wako Pure Chemical Corporation, Japan) and 5 (or 10) mM glucose in Dulbecco’s phosphate-buffered saline (pH 7.4; Nacalai Tesque, Japan). Ag/AgCl (sat. KCl) reference and Pt wire counter electrodes were inserted into the solution. Hydrogel beads containing GOx were then introduced into the chamber and incubated for depositing the beads on the electrode at the bottom. The electrodes were connected to a potentiostat (IviumStat, Ivium Technologies, Netherlands) and placed under an inverted microscope (Nikon, ECLIPSE Ti2, Japan) equipped with an electron-multiplying charge-coupled device camera (ImagEM X2, Hamamatsu Photonics, Japan). The device was incubated within 30 min. The potential for ECL imaging was then stepped from 0 to 1.3 V. The camera exposure started simultaneously after the potential step and ended after 5 s. The ECL images were analyzed using ImageJ software. Briefly, 10 sets of straight lines were drawn radially from the center of the sample to the bulk side, and the gray values of each line were obtained using the Plot Profile function. The flux of the enzymatic product, H_2_O_2_, was calculated using a theoretical equation presented in the Results and Discussion section. The diffusion coefficient of H_2_O_2_ was set as 1.305 × 10^–9^ m^2^/s.^32^ In the previous study, the value was measured using a 0.1 KCl solution.^32^

## Results and Discussion

### Theory

**Figure 1:**
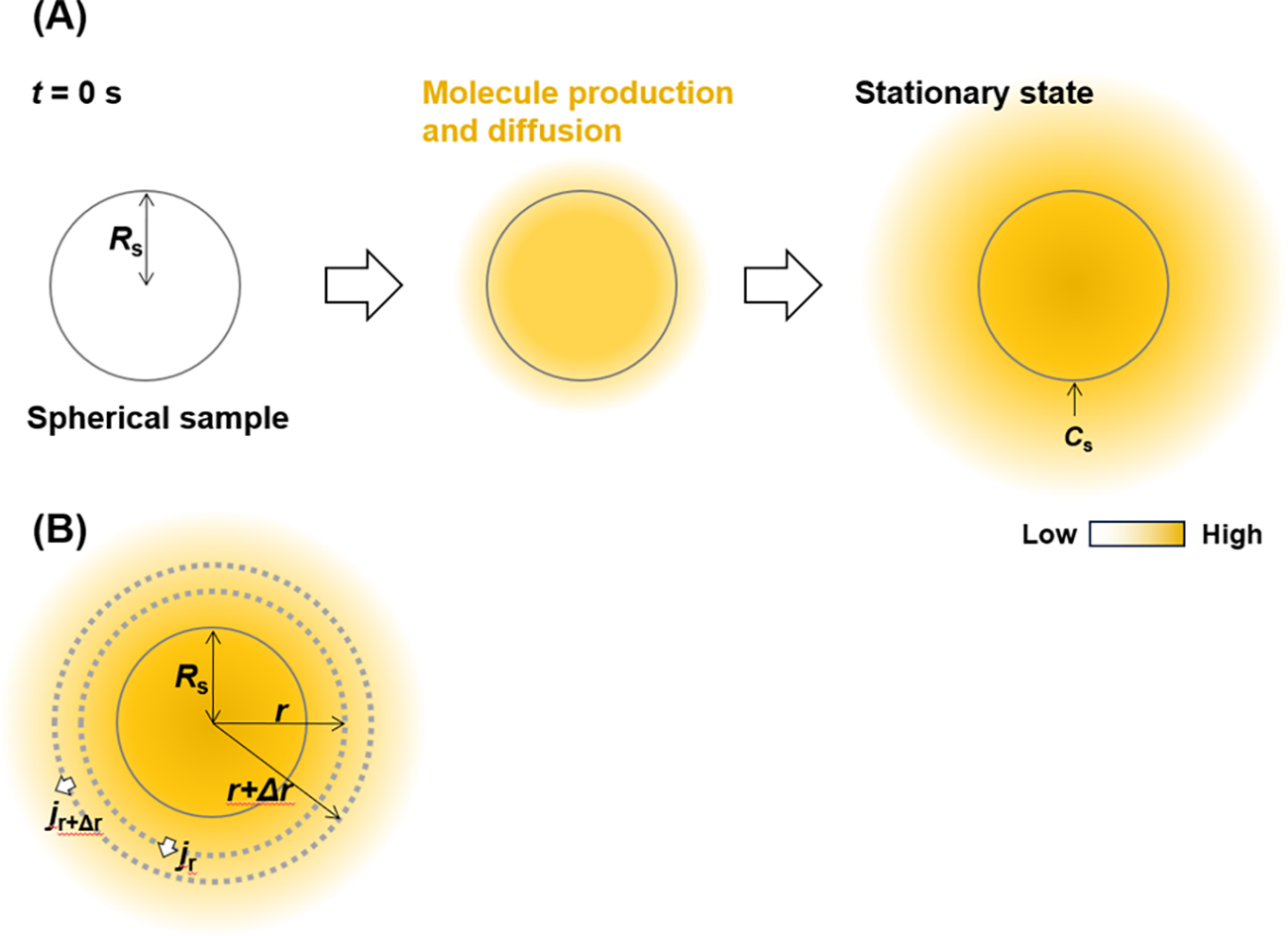
Schematic illustration of the common spherical diffusion theory. (A) Outline of the diffusion model where a spherical sample is set in a solution and produces molecules within the sample. The molecules diffuse to the solution and the model achieves a stationary condition. (B) Outline of the parameters in the theory.

Before discussing the extended spherical diffusion theory, the common spherical diffusion theory is discussed using a radial diffusion model with spherical coordinates (Figure 1A). In this study, constants and variables are written in capital and small letters, respectively. In this theoretical model, a spherical sample is set in a solution without a wall, such as a culture plate bottom. The sample radius is *R*_s_ (m). Molecules are produced within the sample at a constant reaction rate, *K* (mol/(m^3^ s)). The flux of the molecules per a sample in a stationary state, *F* (mol/s), can be expressed as
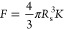
1

Based on this model (Figure 1B), eq 2, which is based on the mass balance through a volume element, 4π*r*^2^Δ*r*, can be obtained (*r ≥ R*_s_):
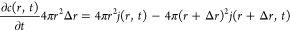
2where *c* is the concentration of the molecules (mol/m^3^), *t* is the time (s), *r* is the distance from the sample center (m), and *j* is the flux density of the molecules (mol/(m^2^ s)) (Figure 1B). The equation can be divided by 4π*r*^2^Δ*r*, yielding
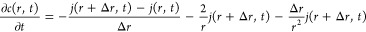
3eq 4 is obtained by taking the limit as Δ*r* → 0:
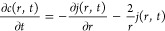
4

According to Fick’s law, eq 5 is obtained:
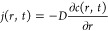
5where *D* is the diffusion coefficient of the molecule (m^2^/s). Combining eqs 4 and 5 yields eq 6:
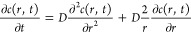
6

By assuming a stationary model, ∂*c*(*r,t*)/∂*t* is zero. Therefore, eq 6 can be reduced to
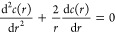
7

The boundary conditions are defined in this production model as follows:
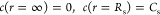
8where *C*_s_ denotes the concentration on the sample surface (mol/m^3^) (Figure 1A) and *c*(*r* = ∞) indicates the concentration at the bulk. Equation 7 was solved using the boundary conditions, resulting in eq 9:
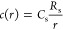
9

Equation 9 can be applied to eq 5 as follows:
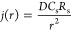
10

According to eq 10, *F* can be expressed as
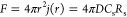
11The values of *C*_s_ and *R*_s_ can be obtained experimentally, allowing *F* to be successfully calculated.

**Figure 2:**
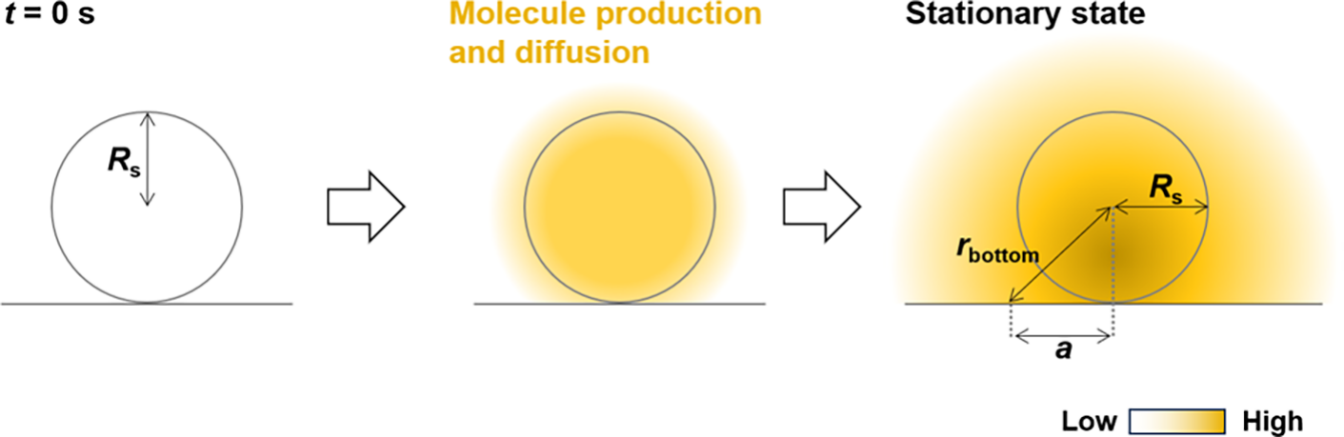
Schematic illustration of the molecules diffusing from a spherical sample on a flat surface. The spherical sample is set on the flat plate and a solution is introduced onto the plate. The sample produces molecules, which diffuse to the solution, and the model achieves a stationary condition.

**Figure 3:**
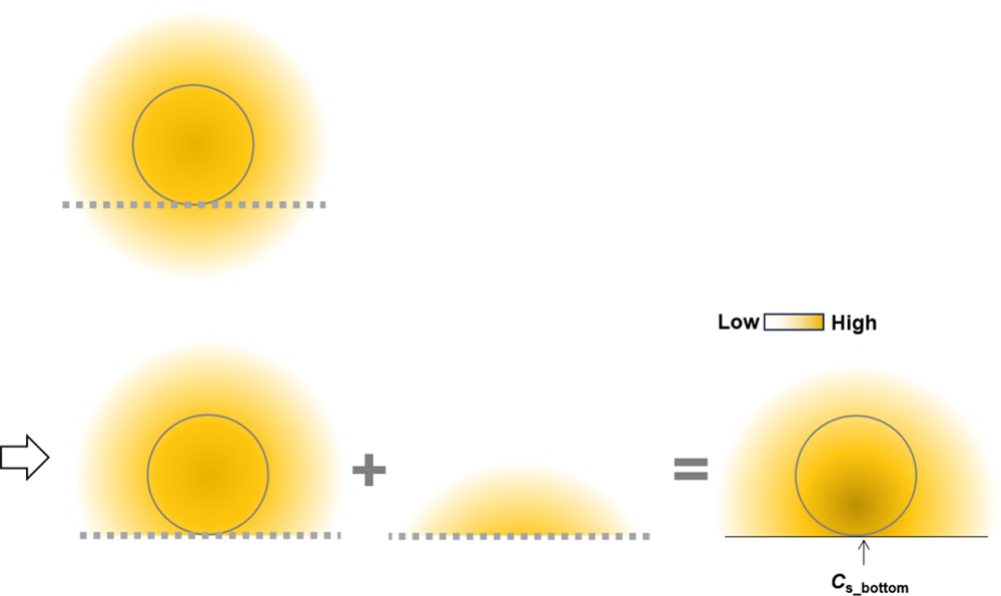
General outline of the extended spherical diffusion theory. When a bottom is set in spherical diffusion, the diffusion is reflected by the bottom, resulting in the sum of the above- and under-concentration profiles, which is the plate model.

In contrast, the samples in most real cases are set on a flat plate and a diffusion layer of molecules is formed on the plate because almost spherical biosamples, such as cells and biobeads, go down to the bottom in assay solutions. Therefore, eq 11 must be modified for the analysis. Figure 2 shows a radial diffusion model with spherical coordinates and a flat plate to extend the spherical diffusion theory. The plate boundary conditions exhibit no flux. *c*_plate_(*r*) is the concentration in this model. *r*_bottom_ is the distance between the center of the sample and the arbitrary position at the bottom (*r*_bottom_*≥ R*_s_), and *c*_plate_ (*r*_bottom_) is the concentration at the bottom. The diffusion process in the model can be expressed as the sum of the processes of individual molecules via individual boundary conditions because they can be considered independent processes (Figure 3). When the plate is set, the molecules do not diffuse over the bottom but affect the boundary conditions at the bottom. The boundary conditions affect the concentration, which indicates that the change is reflected at the bottom. Therefore, the concentration profile obtained using the plate model is the sum of the concentration profiles at the top and bottom of the line indicating the bottom (Figure 3). According to the extended spherical diffusion theory, it was assumed that *c*_plate_(*r*_bottom_) can be obtained using *c*(*r*) in the model without the bottom (eq 9) as follows:
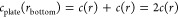
12where the value of *r*_bottom_ equals that of *r*. According to eq 12, the concentration at the sample surface at the bottom, *C*_s_bottom_ (*c*_plate_(*r*_bottom_ = *R*_s_)), can be expressed using *C*_s_ in the model without the bottom (eq 9), as
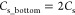
13

Combining eqs 11 and 13 yields
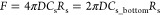
14

**Figure 4:**
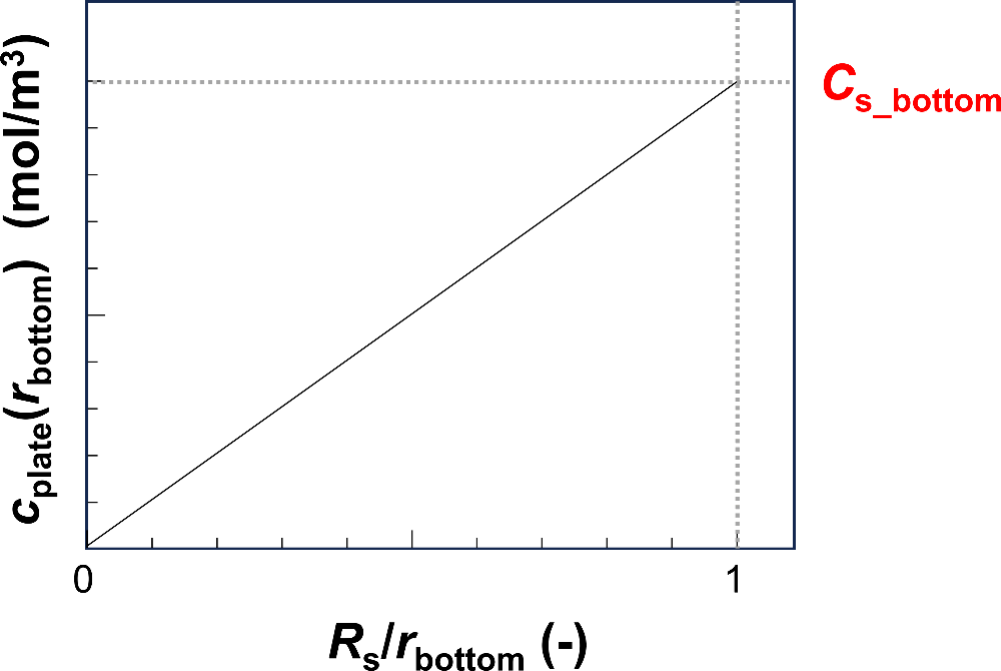
Graph of *c*_plate_(*r*_bottom_) vs *R*_s_/*r*_bottom_ in the plate model. The molecules in this model are produced within the sample. The value of the slope (= *c*_plate_(*r*_bottom_)/(*R*_s_/*r*_bottom_)) indicates *C*_s_bottom_.

Therefore, if *C*_s_bottom_ is obtained experimentally, *F* can be calculated. The ECL imaging approach is one of the choices for obtaining *C*_s_bottom_ because *c*_plate_(*r*_bottom_) can be measured when an electrode is used at the bottom. Under appropriate conditions, the ECL signals (or normalized ECL signals) are proportional to the concentration of the target molecules. In contrast, it is difficult to measure directly *C*_s_bottom_ because biosamples may reflect or consume ECL emissions during ECL imaging. For example, even though *C*_s_bottom_ is directly calculated using the ECL intensity at the bead bottom and the calibration curve, the value might differ from the theoretical value because the ECL intensity at the bead bottom might be affected due to several reasons, such as electrode fouling by the attachment of soft biosamples (e.g., cells and hydrogels). Therefore, *C*_s_bottom_ should be indirectly calculated using the concentration profile at the electrode in ECL imaging. *c*_plate_(*r*_bottom_), which represents the concentration profile at the bottom, can be expressed using eqs 9, 12, and 13:
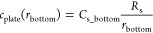
15*c*_plate_(*r*_bottom_) can be obtained using ECL imaging. *R*_s_ and *r*_bottom_ can be obtained using optical microscopy. *r*_bottom_ can be expressed as
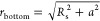
16where *a* is the distance as shown in Figure 2 (*a* ≥ 0). The slope of the *c*_plate_(*r*_bottom_) vs *R*_s_/*r*_bottom_ graph prepared using eq 15 indicates *C*_s_bottom_ (Figure 4). Thus, because *C*_s_bottom_ can be obtained experimentally, *F* can be successfully calculated using the extended spherical diffusion theory and ECL imaging.

**Figure 5:**
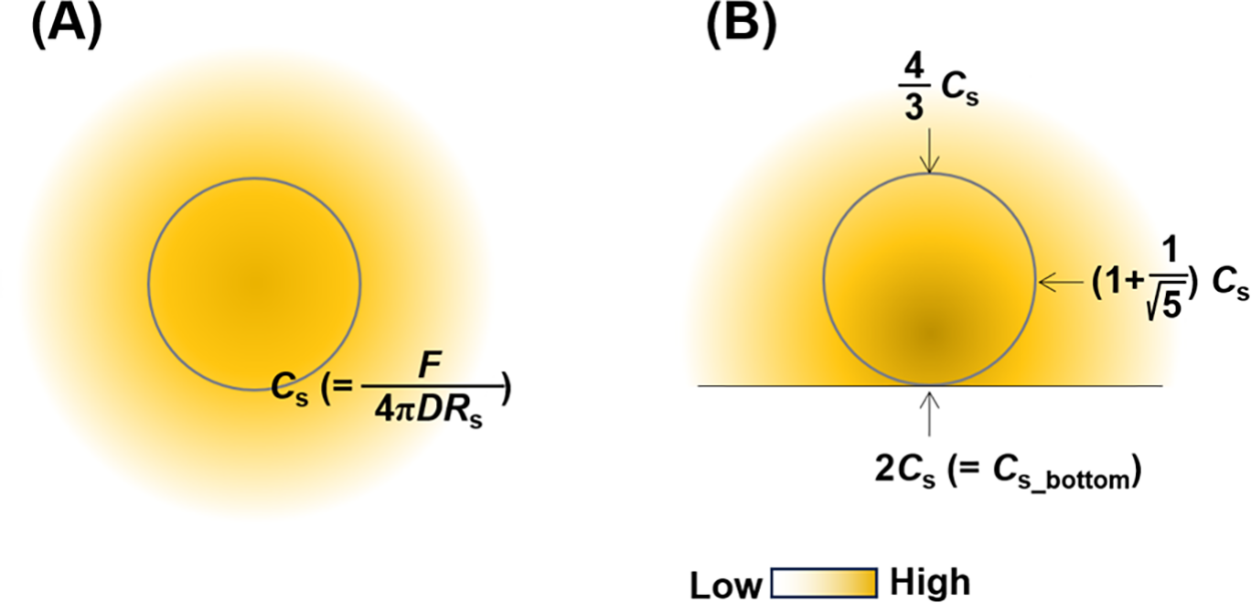
Values of *C*_s_ in the models (A) without and (B) with the plate.

Figure 5 shows the concentrations at the top, bottom, and side of the sample surface in the plate model. Thus, the plate is useful for concentrating molecules. The model can be applied to analyzing the production rate of molecules, such as H_2_O_2_, during enzymatic reactions and cellular activities. Furthermore, this model can be used to analyze the consumption of molecules, such as dissolved oxygen, to evaluate the cellular respiratory activity in a solution. The boundary conditions in the consumption model are
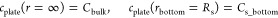
17where *C*_bulk_ is the molecular concentration in the bulk. In the consumption model, eqs 14 and 15 can be respectively modified as
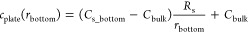
18
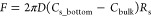
19

**Figure 6:**
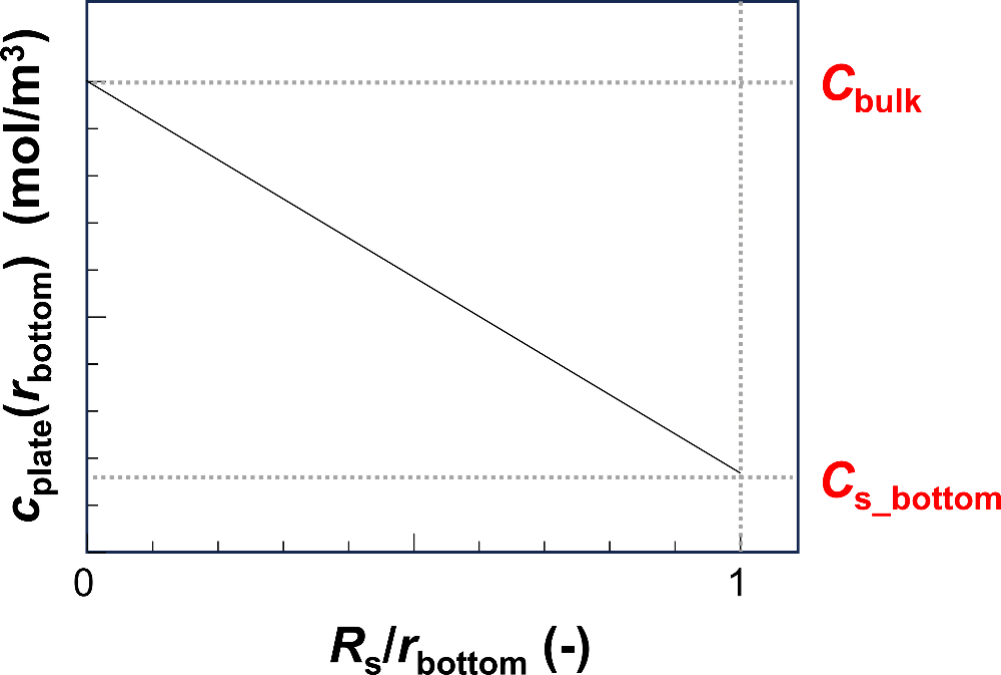
*c*(*r*_bottom_) vs *R*_s_/*r*_bottom_ graph of the consumption of diffusive molecules within a sample. The value of the slope indicates the *C*_s_bottom_ – *C*_bulk_ value.

The slope of the *c*_plate_(*r*_bottom_) vs *R*_s_/*r*_bottom_ graph indicates *C*_s_bottom_ – *C*_bulk_ (eq 18 and Figure 6). As *C*_bulk_ is a known value, *F* can be successfully calculated using the extended spherical diffusion theory and ECL imaging.

**Figure 7:**
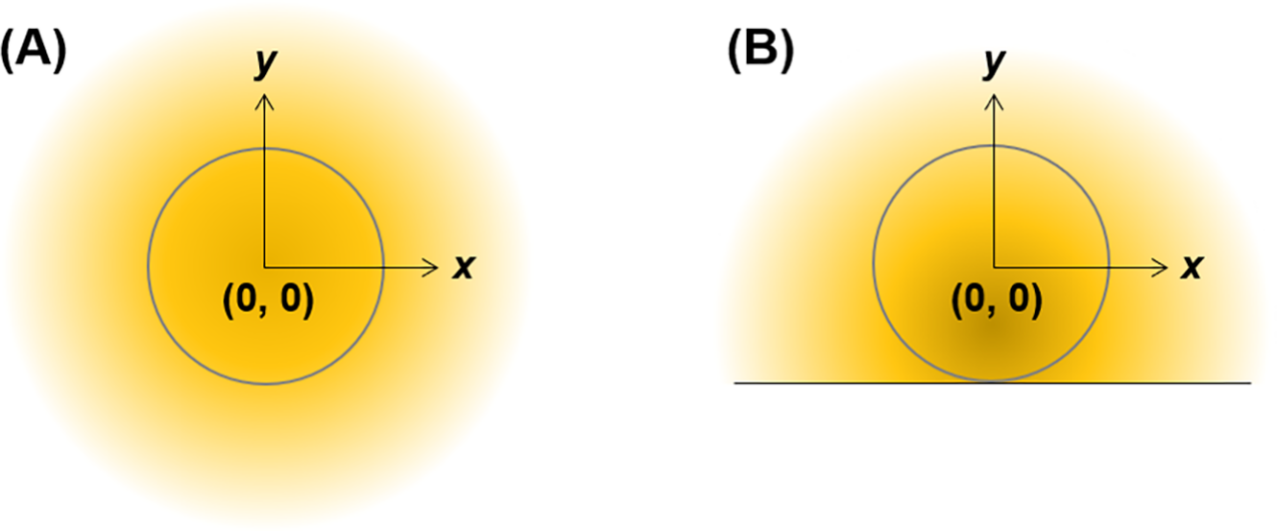
Outline of the *x*- and *y*-axes model for the (A) spherical diffusion and (B) plate models.

The previous paragraph only discusses the concentration profiles at the bottom. This section discusses the concentration profiles over the entire study area. The origin was set as the sample center for the *x*- and *y*-axes (Figure 7). According to the extended spherical diffusion theory, *c*_plate_(*x*,*y*) (*y* ≥ −*R*_s_, *x*^2^ + *y*^2^ ≥ *R*_s_^2^) can be expressed as
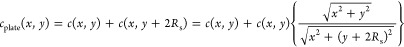
20because of following equations:
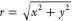
21
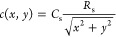
22

Equation 22 is obtained using eqs 9 and 21. Equation 22 can be applied to eq 20, which results in eq 23:
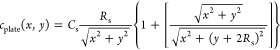
23

eq 23 can be applied to eq 14, which results in eq 24:
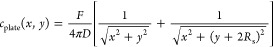
24When *y* equals −*R*_s_, eqs 12–14 can be obtained using eq 24.

**Figure 8:**
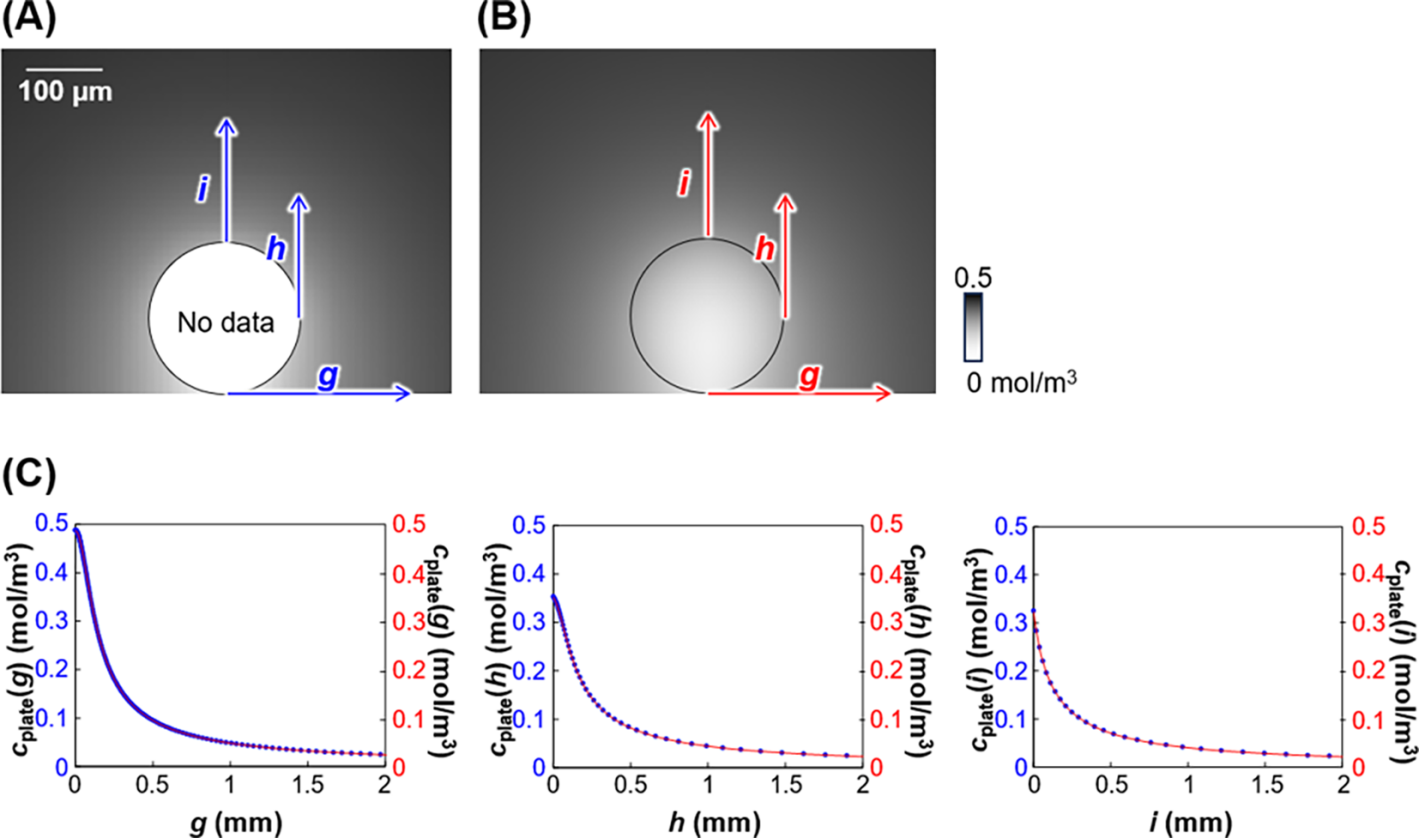
Concentration profiles using (A) theory and (B) the simulation data. eq 24 was used for the theory. The theory has no data for the bead interior because the theory can be used only for *r* ≥ *R*_s_. (C) *c*_plate_ vs *g*, *h*, and *i*, using the theoretical and simulation models. The blue and red data represent theory and the simulation data, respectively. *g* is the horizontal distance from the sample bottom (*x* = 0, *y* = −*R*_s_). *g* is the same as *a*. *h* and *i* are the vertical distances from the sample side (*x* = *R*_s_, *y* = 0), and top (*x* = 0, *y* = *R*_s_), respectively. *g*, *h*, and *i* ≥ 0.

A simulation was subsequently performed using the finite element method, and the values were compared with those obtained theoretically to validate the theory. As shown in Figures 8A and 8B, the simulated concentration profile was the same as that obtained theoretically. Horizontal and vertical concentration profiles at the bottom and from the top and sides of the sample surface, respectively, were prepared for a detailed comparison (Figure 8C). Each simulated profile was the same as that theoretically derived. Thus, the theory successfully expressed the concentration profiles of diffusive molecules from a biosample on a flat surface. Because this theory can express concentrations over an entire area, it can be used for SECM analyses. The analysis results are discussed in Figure S3.

**Figure 9:**
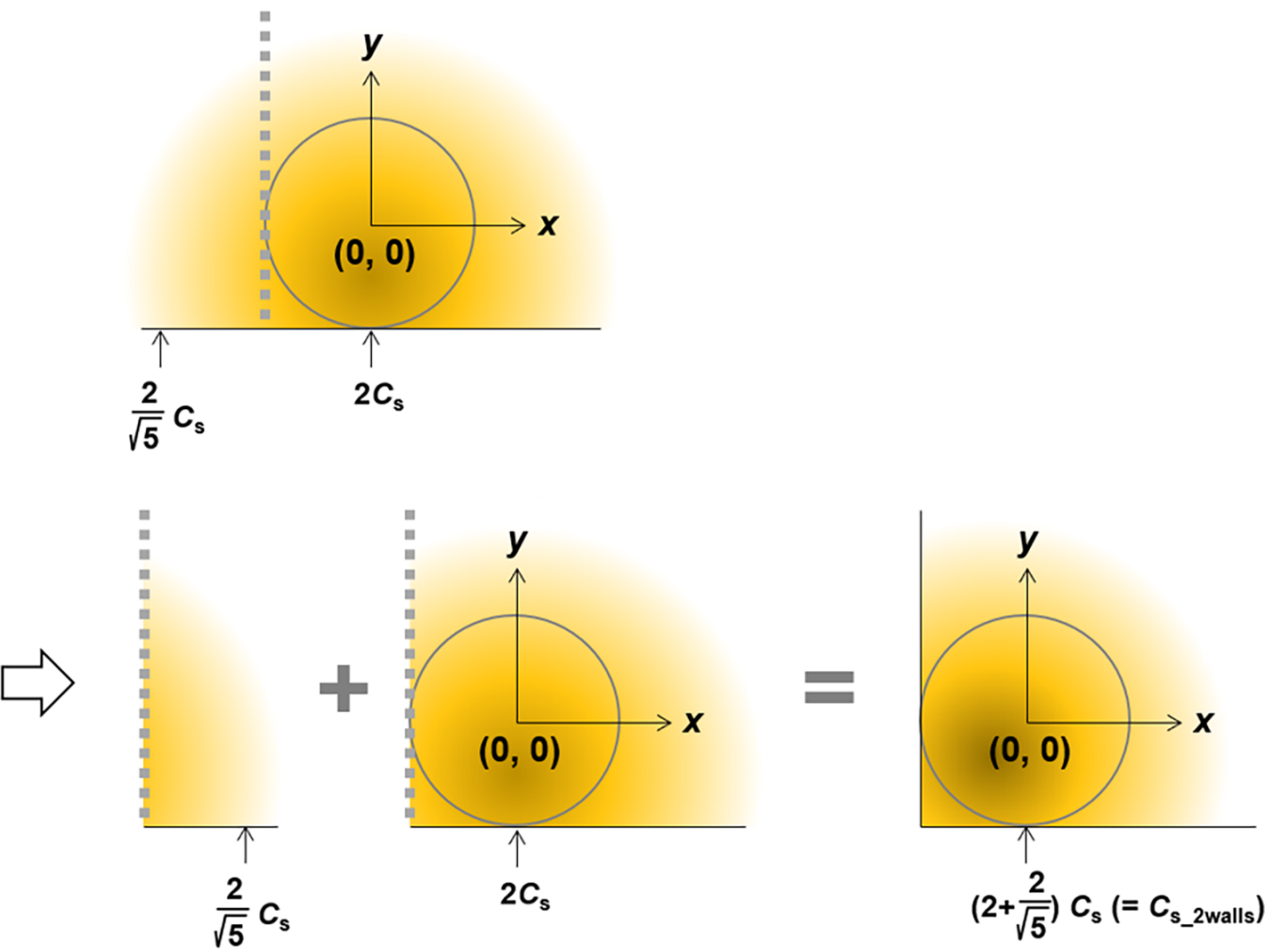
Outline of the two-wall model using the extended spherical diffusion theory. When an additional wall is set in the previous extended spherical diffusion model shown in Figure 3, the diffusion is further reflected by the wall, resulting in the sum of the concentration profiles.

The extended spherical diffusion theory can be applied to expressing the concentration profile when an additional wall is used, as shown in Figure 9. The concentration in the two-wall model can be expressed as the sum of the concentration profiles via the additional wall (Figure 9). The concentration is defined as *c*_2walls_(*x*,*y*). According to the extended spherical diffusion theory, *c*_2walls_(*x*,*y*) can be expressed as
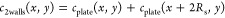
25

Equation 25 can be applied to eq 24, which results in eq 26:
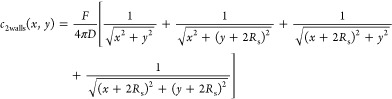
26

**Figure 10:**
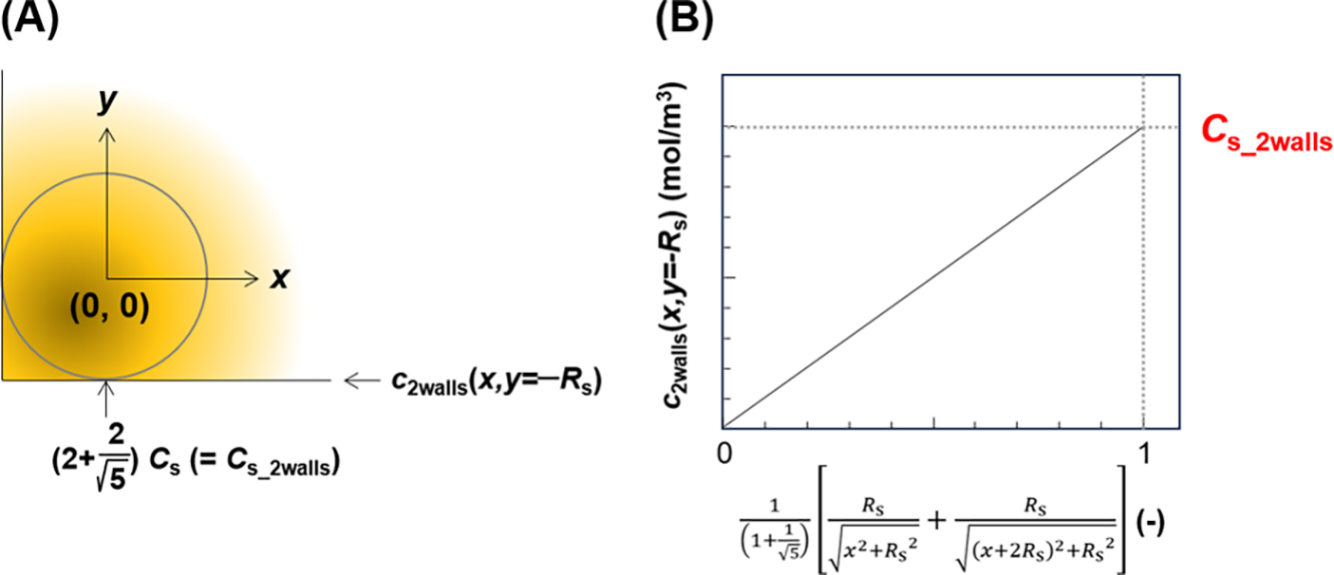
Concentration profiles in the two-wall model. (A) General outline of the model and (B) graph for calculating *C*_s_2walls_.

When *x* = 0 and *y* = −*R*_s_, *C*_s_2walls_, as shown in Figure 10A, can be expressed as
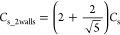
27*c*_2walls_(*x*,*y* = −*R*_s_) can be expressed as
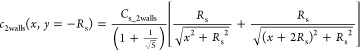
28

Because the values of *c*_2walls_(*x*,*y* = −*R*_s_), *R*_s_, and *x* can be experimentally obtained, *C*_s_2walls_ can be calculated from the graph shown in Figure 10B. *F* can be obtained as follows:
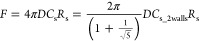
29

Thus, the extended spherical diffusion theory is useful for expressing the concentration profiles of the diffusive molecules in a spherical sample surrounded by two flat walls.

### ECL Imaging of Hydrogel Beads Containing GOx

**Figure 11:**
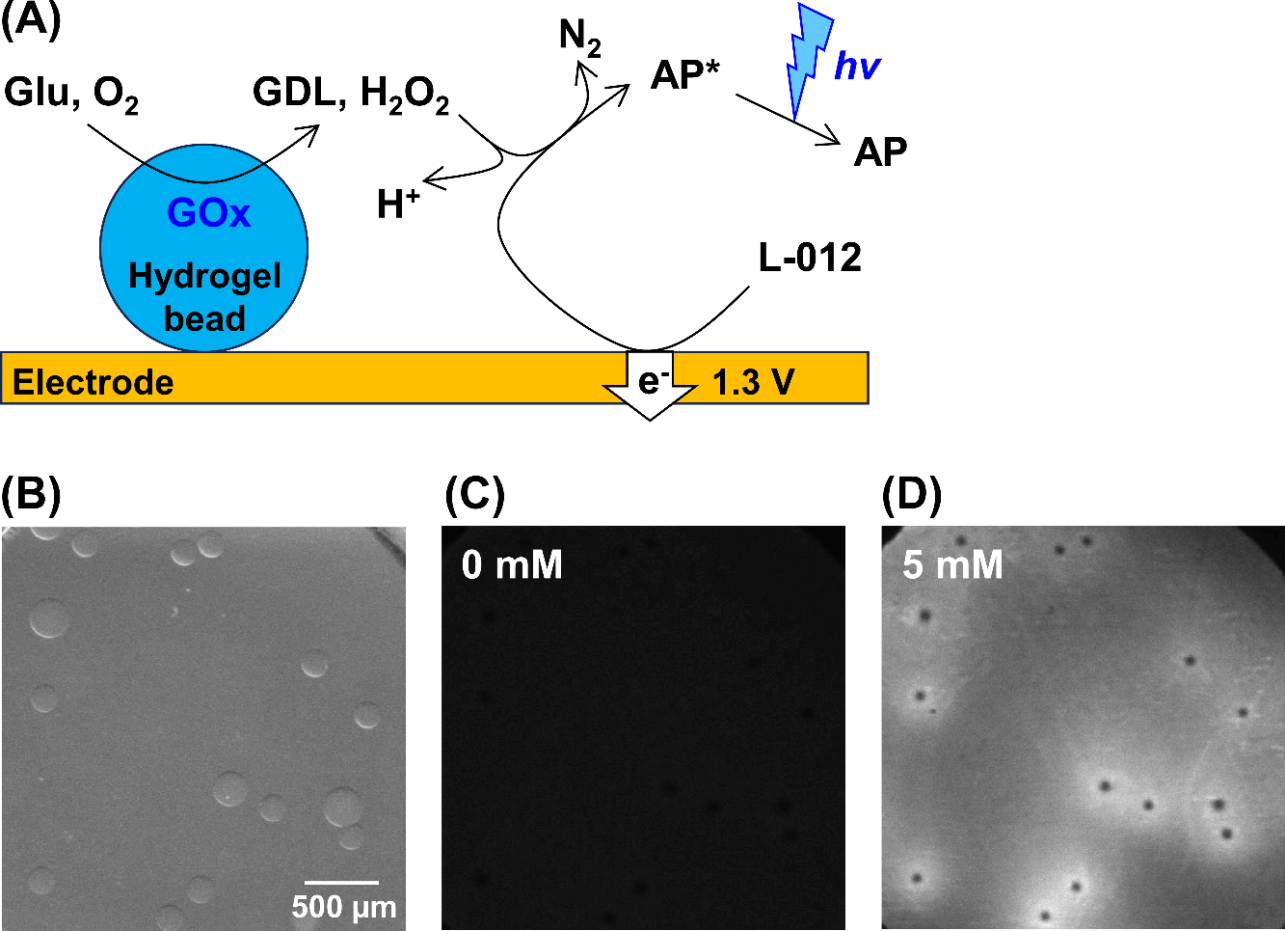
ECL imaging of H_2_O_2_ from hydrogel beads containing GOx. (A) Schematic illustration. One of the ECL reactions using L-012 is shown. [Legend: Glu, glucose; GDL, glucono-δ-lactone (gluconolactone); AP, 3-aminophthalate species; AP*, excited state of AP.] (B) Bright-field image of the hydrogel beads. (C, D) ECL images of the hydrogel beads in 0 mM glucose (panel (C)) and 5 mM glucose (panel (D)).

For the proof-of-concept experiments, hydrogel beads containing GOx were measured using ECL imaging, and the flux of enzymatic products was analyzed using the extended spherical diffusion theory. Figure 11A shows the ECL imaging detection scheme. The beads produce H_2_O_2_ in the presence of glucose, because of the enzymatic reaction with GOx. The H_2_O_2_ diffuses to form a H_2_O_2_ diffusion layer around the beads. L-012 oxidizes upon the application of 1.3 V. The resulting chemical reacts with H_2_O_2_ to produce AP*, and luminescence is obtained on the electrode. Figure 11B shows a bright-field image of the beads on the electrode. The bead radius was 120.4 ± 15.5 μm. In the absence of glucose, no ECL signal was observed because H_2_O_2_ was not produced (Figure 11C). In contrast, ECL signals were observed around the beads in the presence of glucose (Figure 11D), and a H_2_O_2_ diffusion layer was observed. Thus, ECL imaging can rapidly measure many samples, indicating that imaging is advantageous for SECM. Its spatial resolution is superior to that of conventional electrode arrays; therefore, ECL imaging is suitable for analyzing the concentration profile of each small biosample. Figure 11D also shows that there was no ECL signal at the center of the bead, indicating that the beads might have blocked the diffusion of glucose and/or L-012 to the electrode, and that the electrode might have been fouled by the beads. Therefore, the ECL signals under the beads were not used to analyze the flux in the following discussion.

**Figure 12:**
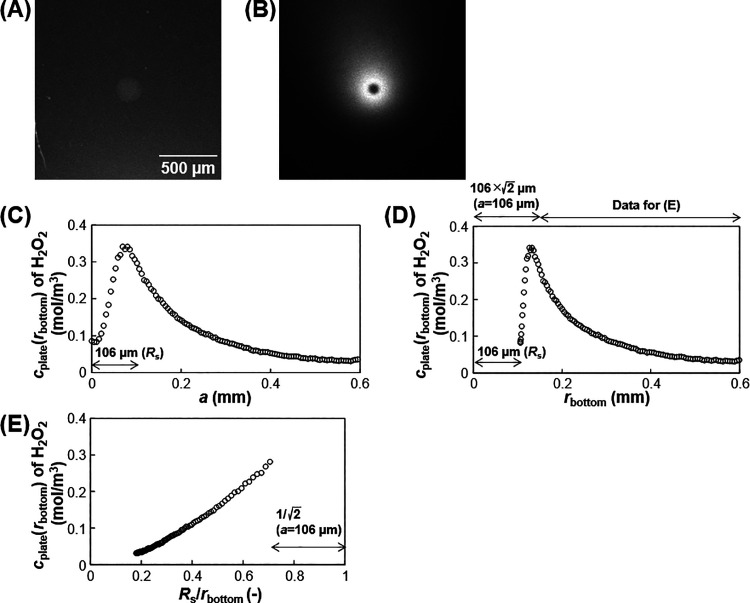
Analysis of the H_2_O_2_ diffusing from a single hydrogel bead. (A) Bright-field and (B) ECL images in 10 mM glucose. The radius of the bead (*R*_s_) was 106 μm. (C) *c*_plate_(*r*_bottom_) of H_2_O_2_ vs *a*. (D) *c*_plate_(*r*_bottom_) of H_2_O_2_ vs *r*_bottom_. (E) *c*_plate_(*r*_bottom_) of H_2_O_2_ vs *R*_s_/*r*_bottom_. The data of *a* ≥ 106 μm was plotted into the graph to eliminate the effect of quenching the ECL at the bead center.

**Figure 13:**
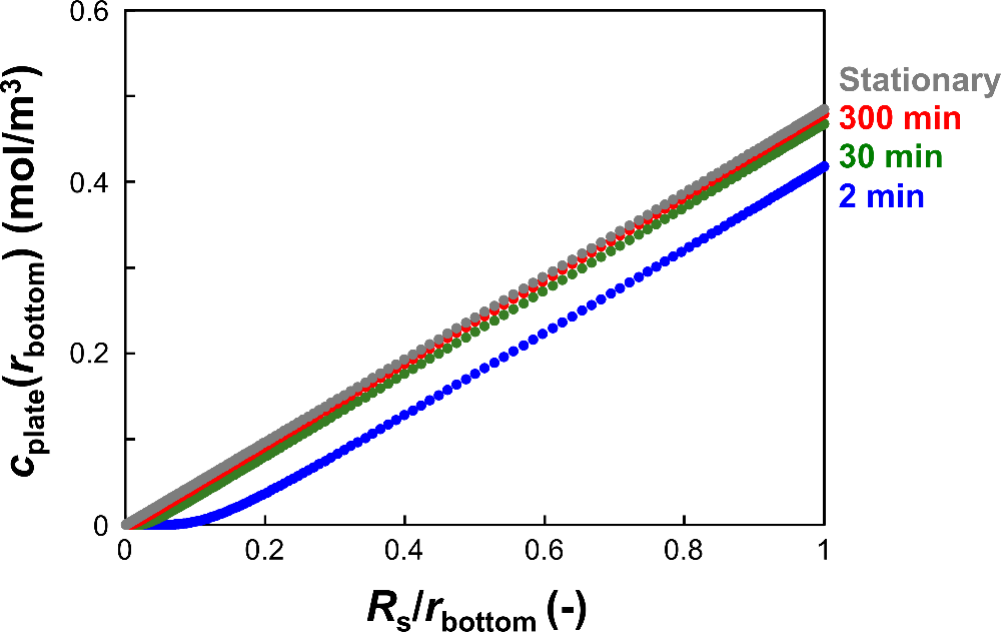
Simulation results of the time-dependent models. The graph shows *c*_plate_(*r*_bottom_) vs *R*_s_/*r*_bottom_ at 2, 30, and 300 min. The result of the stationary model is also included in the graph.

Figure 12 shows the analysis results of the GOx beads using the extended spherical diffusion theory. Figures 12A and 12B show the representative bright-field and ECL images, respectively. The radius of the beads was 106 μm. *r*_bottom_ and *a*, as shown in Figure 2, were used for this analysis. The ECL signals were converted to H_2_O_2_ concentrations using a calibration curve (Figure S4). Figures 12C and 12D show the H_2_O_2_ concentration profiles at the electrode. A *c*_plate_(*r*_bottom_) vs *R*_s_/*r*_bottom_ graph was prepared to compare the profile with that obtained theoretically (Figure 12E). Although the intercept in the theoretical model is zero because *C*_bulk_ is 0 mol/m^3^ (Figure 4), the experimental data graph shows that the intercept is not zero (Figure 12E). According to the graph, *c* is zero when *r*_bottom_ is ∼740 μm, indicating that the thickness of the H_2_O_2_ diffusion layer was 740 μm. The results also indicated that the diffusion layer stopped growing, because of convection and/or an insufficient incubation time to reach the stationary state. Therefore, a time-dependent diffusion simulation was conducted to fill the gap between theoretical and experimental results. As shown in Figure 13, more than 300 min were required to achieve stationary conditions. However, the slope indicating *C*_s_bottom_ in the stationary model was almost the same as that in the time-dependent models at 2 and 30 min, even though the diffusion layer grew during these periods. These results indicate that the slope of the experimental results (Figure 12E) can be used to calculate *C*_s_bottom_ even though the experimental conditions are not stationary. The slope of the experimental data was determined to be 0.45 mol/m^3^. Therefore, *F* was calculated using eq 14 to be ∼400 fmol/s. This value is similar to that of the respiratory activity of cellular spheroids. Thus, this approach can be applied to the analysis of cellular respiratory activity.

Although fiber samples were previously analyzed using simulated results,^33^ the extended diffusion theory will be applied for the analysis.

ECL imaging was performed in this study to validate the extended spherical diffusion theory because ECL imaging can measure the concentration profile on a flat electrode with spherical biosamples. In addition to ECL imaging using a single working electrode, ECL and fluorescence imaging using bipolar electrode (BPE) arrays can also be used to measure the concentration profile on a flat surface.^34−38^ Close BPE arrays can separate biosamples with an ECL chemical solution, which is advantageous, compared to the direct ECL imaging. Previously, the cellular respiratory activity has been successfully visualized using ECL imaging based on BPE arrays.^39^ The extended spherical theory is expected to be used in such imaging systems.

## Conclusions

This study proposes the analysis of diffusive molecules from spherical biosamples on a flat surface. A novel theory called the extended spherical diffusion theory was proposed for this purpose. The simulation results confirmed that the theory successfully expresses the concentration profiles on a flat surface. Hydrogel beads containing GOx were introduced onto an electrode, and the enzymatic product (H_2_O_2_) was visualized on the electrode using ECL imaging. Although the experimental and theoretical thickness of the diffusion layer did not completely correspond, the slope value in the graph did not change, even though the thickness of the diffusion layer changed. These results indicate that the slope of the experimental data is important for analyzing *F*. Thus, this study presents novel guidelines for this analysis. Our proposed approach can be used to evaluate digital immunoassays and engineered organs.

## Supporting Information Available

Acknowledgments, simulation model (Figure S1), device outline (Figure S2), theory for SECM analysis (Figure S3), calibration curve of H_2_O_2_ (Figure S4) (PDF)
