# Supplementary material for: Extended Spherical Diffusion Theory: Electrochemiluminescence Imaging Analysis of Diffusive Molecules from Spherical Biosamples
Source: Anal Chem. 2024 Nov 19;96(48):18967–76. doi: 10.1021/acs.analchem.4c03167 (PMC11618750; doi:10.1021/acs.analchem.4c03167)
Supplement: Supplementary file 1 — ac4c03167_si_001.pdf [file ac4c03167_si_001.pdf]

Supporting information for

Extended spherical diffusion theory: Electrochemiluminescence imaging analysis of diffusive molecules from spherical biosamples

Kosuke Ino[1,*], Miyu Mashiko[1], Yusuke Kanno[2], Yeyi Tang[3], Shuzo Masui[4], Takasi Nisisako[2], Kaoru Hiramoto[5], Hiroya Abe[1,5], Hitoshi Shiku[1,6,**]

[1] Graduate School of Engineering, Tohoku University, 6-6-11-604 Aramaki-aza Aoba, Aoba-ku, Sendai 980-8579, Japan

[2] Institute of Integrated Research, Institute of Science Tokyo, Yokohama 226-8503, Japan

[3] Department of Mechanical Engineering, School of Engineering, Institute of Science Tokyo, Tokyo 152-8550, Japan

[4] Department of Precision Engineering, The University of Tokyo, Hongo 7-3-1, Bunkyo-ku, Tokyo 113-8656, Japan

[5] Frontier Research Institute for Interdisciplinary Sciences, Tohoku University, Aramaki-aza Aoba 6-3, Aoba-ku, Sendai 980-8578, Japan

[6] Graduate School of Environmental Studies, Tohoku University, 6-6-11-604 Aramaki-aza Aoba, Aoba-ku, Sendai 980-8579, Japan

*,** Corresponding authors

E-mail addresses: kosuke.ino@tohoku.ac.jp (K. I.) and hitoshi.shiku.c3@tohoku.ac.jp (H. S.)

Contents

-Acknowledgments

-Figure S1: Simulation model

-Figure S2: Device outline

-Figure S3: Theory for SECM analysis

-Figure S4: Calibration curve of H_2O_2

Acknowledgments

This work was supported by a Grant-in-Aid for Early-Career Scientists (Nos. 22K14707 and 24K17706), a Grant-in-Aid for Scientific Research (A) (No. 20H00619), a Grant-in-Aid for Scientific Research (B) (Nos. 21H01957 and 22H02102), a Grant-in-Aid for Challenging Research (Exploratory) (No. 23K17926), and a Grant-in-Aid for Scientific Research (S) (No. 24H00070) from the Japan Society for the Promotion of Science. This study was supported by Amano Institute of Technology, and the Precise Measurement Technology Promotion Foundation. This study was partially supported by AMED (Grant Numbers JP22be1004205, JP23be1004205, and JP24be1004205). This work was also supported by JST COI (No. JPMJCE1303) from the Japan Science and Technology Agency.

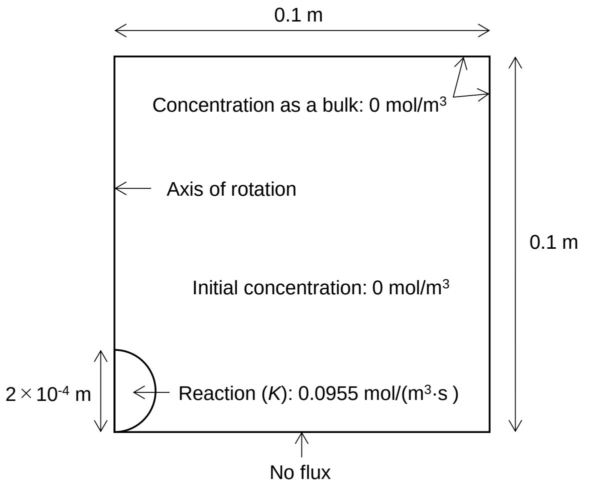

Other parameters and conditions

Diffusion coefficient (D) : 1.305×10^{-9} m^2/s

Chemical species transport: transport of diluted species

General studies: stationary (or time dependent for Fig. 13)

Mesh size: extremely fine

Figure S1

Simulation model showing the initial and boundary conditions, parameters, and conditions. As the reaction (K) and radius (R_s) of the bead were set to be 0.0955 mol/(m^3·s) and 100 µm, respectively, F (flux per a sample) was calculated to be 400 fmol/s using Equation 1. Not to scale.

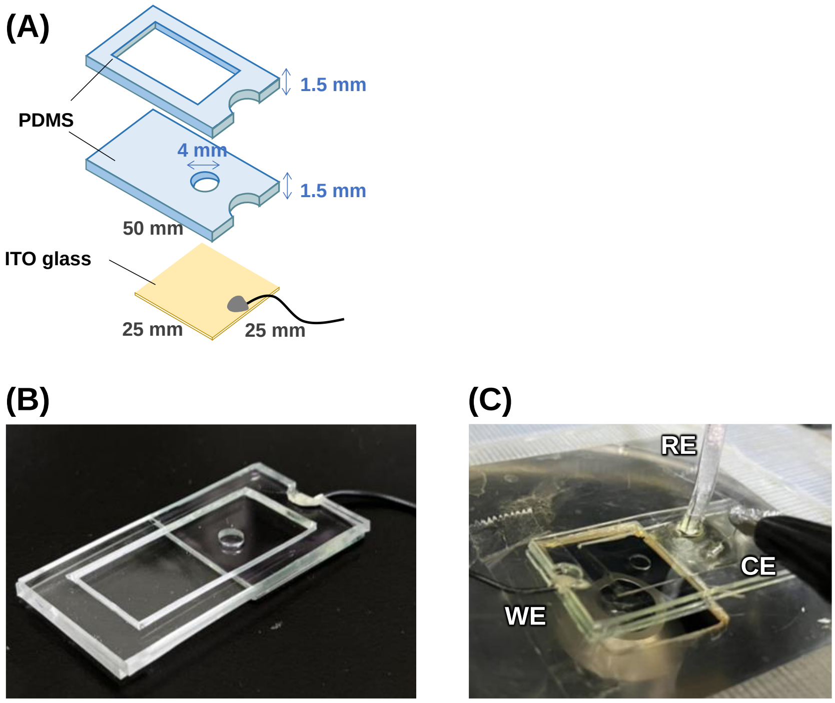

Figure S2

Outline of the device. (A) Fabrication scheme. (B) Image of the device. (C) Image of the electrochemical cell. WE: working electrode. RE: reference electrode. CE: counter electrode. ITO: indium tin oxide.

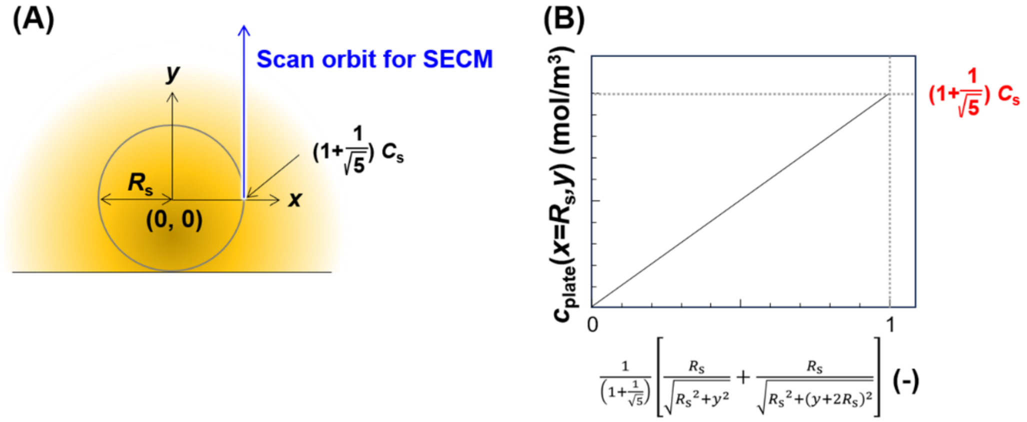

Figure S3

SECM analysis using the extended spherical diffusion theory. (A) Schematic illustration of the model in which a probe is vertically scanned from the side surface. (B) Graph for calculating C_s. Equation S1 can be used for the SECM analysis.

$$c_{\mathrm{plate}}(x = R_s, y) = C_s\left[\frac{R_s}{\sqrt{R_s{}^2+y^2}} + \frac{R_s}{\sqrt{R_s{}^2+(y+2R_s)^2}}\right] \qquad \text{(S1)}$$

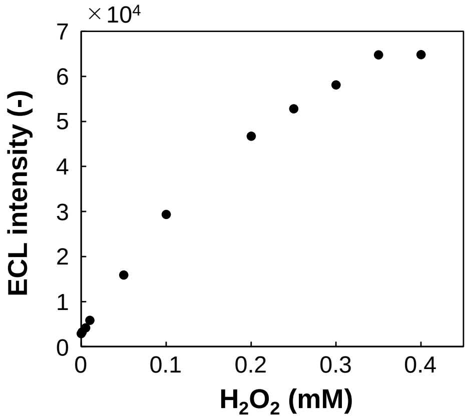

Figure S4

ECL intensity vs H_2O_2 concentration for the calibration curve.
